# Genomic Characterisation of Antibiotic-Resistant *Escherichia coli* from an Intensive Poultry Production System in the uMgungundlovu District, KwaZulu-Natal, South Africa: A Snapshot

**DOI:** 10.3390/antibiotics15020174

**Published:** 2026-02-05

**Authors:** Nelisiwe S. Gumede, Joshua Mbanga, Charles Hunter, Melissa Ramtahal, Sabiha Y. Essack, Linda A. Bester

**Affiliations:** 1 Antimicrobial Research Unit, College of Health Sciences, University of KwaZulu-Natal, Durban 4001, South Africa; gumedenelly0@gmail.com (N.S.G.); joshmbanga@gmail.com (J.M.); ramtahal@ukzn.ac.za (M.R.); essacks@ukzn.ac.za (S.Y.E.); 2 Department of Applied Biology and Biochemistry, National University of Science and Technology, Bulawayo P.O. Box AC939, Zimbabwe; 3 Discipline of Microbiology, School of Life Sciences, College of Agriculture, Engineering and Science, University of KwaZulu-Natal, Pietermaritzburg 3209, South Africa; hunterc@ukzn.ac.za; 4 School of Pharmacy, University of Jordan, Amman 11942, Jordan; 5 Biomedical Resource Unit, School of Medicine, College of Health Sciences, University of KwaZulu-Natal, Durban 4001, South Africa

**Keywords:** *Escherichia coli*, antibiotic resistance, whole genome sequencing, poultry production, South Africa

## Abstract

**Background**: Intensive poultry production systems can act as reservoirs for antibiotic-resistant and multidrug-resistant (MDR) *Escherichia coli*, posing a public health risk through food and environmental transmission. **Methods:** This study investigated the genomic characteristics of antibiotic-resistant *E. coli* isolated from an intensive poultry production system in the uMgungundlovu District, KwaZulu-Natal, South Africa. Chicken litter, wastewater, and floor swab samples were collected over three consecutive production cycles. Putative *E. coli* isolates were detected using the Colilert-18 system, cultured on eosin methylene blue agar, and genomically confirmed by quantitative PCR (q-PCR) targeting the *uidA* gene. Whole genome sequencing was performed using the Illumina MiSeq platform, followed by bioinformatic analyses to assess resistance genes, mobile genetic elements, and phylogenetic relationships. **Results**: Of 150 presumptive *E. coli*, 70 were genomically confirmed as *E. coli* and resistant to at least one antibiotic, with 74% exhibiting multidrug resistance. Resistance was highest to tetracycline (100%), ampicillin (94%), and trimethoprim–sulfamethoxazole (76%), while ciprofloxacin resistance was rare (3%). Genomic analysis identified multiple antibiotic resistance genes conferring resistance to fluoroquinolones, β-lactams, aminoglycosides, amphenicols, fosfomycin, and sulfonamides, as well as the disinfectant resistance gene *qacI*. These genes were frequently associated with mobile genetic elements, including plasmids, integrons, transposons, and insertion sequences. Predominant sequence types included ST155, ST48, ST1286, and ST602, with phylogenetic relatedness to poultry-associated isolates from Cameroon, Ghana, Nigeria, and Tanzania, as well as environmental *E. coli* strains previously identified in South Africa and Ghana. **Conclusions**: The detection of diverse, mobile MDR *E. coli* lineages in poultry environments clearly signals a substantial risk for resistance gene dissemination into the food chain and surrounding ecosystems. Immediate attention and intervention are warranted to mitigate public health threats.

## 1. Introduction

*Escherichia coli* (*E. coli*) is a commensal bacterium commonly found in the gastrointestinal tract of warm-blooded animals [1]. It is widely used as an indicator organism because it is well characterised and relatively easy to isolate and study [2]. The detection of *E. coli* in food products and water is considered an indication of faecal contamination, which supports its use in monitoring hygiene and water quality [3]. Although *E. coli* is typically harmless, certain strains can become pathogenic under favourable conditions, causing disease that may require antibiotic treatment [4]. The excessive and inappropriate use of antibiotics contributes to the emergence of antibiotic-resistant bacteria, potentially rendering these treatments ineffective in the future [5].

A recent scoping review reported an increasing prevalence of antibiotic-resistant *E. coli* across Africa in both clinical and non-clinical settings, with prevalence rates reaching up to 98.3% [6]. High levels of resistance were observed against commonly used antibiotics, including ampicillin, tetracycline, and trimethoprim–sulfamethoxazole, across several African countries [6]. Similarly, a review by Ramtahal et al. [7] documented widespread resistance within the poultry industry to ampicillin, tetracycline, and sulfonamides, as well as to fluoroquinolones and third-generation cephalosporins. In the poultry industry, antibiotics are used for growth promotion, disease prophylaxis, and metaphylaxis and are typically administered orally at varying doses depending on their purpose [8].

The use of antibiotics in the intensive poultry production industry significantly enhances chicken growth rates, reduces disease prevalence, and provides benefits to both breeders and their flocks [9]. However, administering low doses of antibiotics to chickens for growth promotion creates selective pressure, which leads to the development of antibiotic-resistant bacteria [10]. The transmission of antibiotic resistance genes is facilitated by mobile genetic elements (MGEs), including integrons, insertion sequences, plasmids, and transposons [11]. Antibiotic-resistant *E. coli* has become a significant concern in intensive poultry farming, as it is frequently linked to animal disease, food safety risks, and public health implications [12]. Pathogenic strains such as avian pathogenic *E. coli* (APEC) are responsible for colibacillosis are found associated with poultry, and this leads to increased mortality in poultry and significant economic losses within the poultry industry [1].

The loss in profit puts a strain on the economy of low- and middle-income countries like South Africa, since the poultry industry is among the major contributors to agricultural revenue, accounting for 18% of the total agricultural gross value [10]. Recent data from the Department of Health, South Africa, reported a marked increase in antibiotic importation for animal use, reaching 2,488,754 tons and nearly doubling the amount used for human disease [13]. The spread of antibiotic-resistant *E. coli* is facilitated by such use, with potential dissemination through both animal products and the environment.

Antibiotic-resistant bacterial strains potentially spread to humans through occupational exposure or via the food chain [8,14]. Additionally, the transfer of antibiotic-resistant bacteria to soil and aquatic environments has been demonstrated through runoff of chicken litter used as fertilisers [15]. To gain a deeper understanding of *E. coli*’s genetic makeup and mechanisms of antibiotic resistance, next-generation sequencing (NGS) is essential. Whole genome sequencing (WGS) is particularly valuable as it provides detailed information on the bacterium’s genome, including pathogenicity and virulence factors [4].

To assess the prevalence and dissemination of antibiotic-resistant *E. coli* in an intensive poultry production system, *E. coli* genomes from chicken litter, wastewater, and floor swabs were characterised using whole genome sequencing and bioinformatics analysis to delineate the resistome, mobilome, and phylogeny.

## 2. Results

### 2.1. Prevalence of E. coli and Antibiotic Susceptibility Profiling

Out of 54 samples collected, 150 bacterial isolates were randomly selected from chicken litter and wastewater samples to constitute the final sample size, and 47% (*n* = 70) were confirmed as *E. coli* through molecular screening. Moreover, *E. coli* was not isolated from any of the floor swab samples. All confirmed *E. coli* isolates exhibited resistance to at least one antibiotic. Notably, all isolates were resistant to tetracycline (TET). Additionally, 94% (*n* = 66) showed resistance to ampicillin (AMP), while 76% (*n* = 53) were resistant to trimethoprim–sulfamethoxazole (SXT). The lowest resistance rate was observed against ciprofloxacin (CIP), in only 3% (*n* = 2) of isolates. No resistance was detected to gentamicin (GEN), meropenem (MEM), cefotaxime (CTX), ceftriaxone (CRO), ceftazidime (CAZ), or cefepime (FEP) (Table 1). The resistant *E. coli* isolates consistently exhibited similar resistance profiles across all sampling cycles, except for cycle 2 (December), during which two isolates showed resistance to ciprofloxacin (Table 1). Ciprofloxacin also had the highest proportion of intermediate susceptibility among the antibiotics tested, with 23% (*n* = 16) of isolates falling into this category. Multidrug resistance (MDR) was defined as, i.e., microbes, resistant to at least one antibiotic in three or more classes [16]. A total of 74% (*n* = 52) isolates were classified as MDR. The most common antibiogram pattern, AMP-SXT-TET, accounted for 71% (*n* = 50). AMP-TET-SXT-CIP and AMP-TET-CIP accounted for 1.4% (*n* = 1) each, as shown in Table 1.

### 2.2. Escherichia coli Antibiotic Resistance Genes

The whole genome analysis (Table 2) demonstrated that a higher number of isolates, 68% (*n* = 17), carried fluoroquinolone resistance genes in the genotypic analysis compared to the phenotypic AST results. The most frequently detected fluoroquinolone resistance gene was *qnrS1*, followed by *oqxA* and *oqxB.* One isolate (EC04) carried *qnrB19*, which is linked to plasmid-mediated quinolone resistance. Out of 17 isolates carrying fluoroquinolone resistance genes, eight isolates represented chicken litter, and the remaining nine represented wastewater. A total of 72% (*n* = 18) isolates carried the disinfectant gene *sitABC*, and 52% (*n* = 13) of *sitABC* genes were carried on isolates harbouring fluoroquinolone resistance.

Extended-spectrum β-lactamase (ESBL) genes were also identified, with multiple *bla_TEM_* genes detected. *Bla_TEM1B_* was the most common gene, followed by *bla_TEM_-_135_* and the *bla_LAP-1_* gene. A total of 36% (*n* = 9) of isolates carried ESBL-resistant genes, with a distribution of four isolates from chicken litter and five from wastewater. These results correspond with the AST results. Other resistance genes identified included those conferring resistance to aminoglycosides (*aadA1, aadA2*, *aadA5*, *aph(3′)-Ia*, *aph(3″)-Ib*, *aph(6)-Id*), bleomycin (*bleo*), chloramphenicol (*cmlA1*), fosfomycin (*fosA3, fosA4*), sulfamethoxazole (*sul2*, *sul3*), tetracycline (*tetA, tetB, tetM*), and trimethoprim (*dfrA5, dfrA12, dfrA14, dfrA15*, *dfrA17*). Isolates were further investigated for mutations in quinolone resistance determinant regions (QRDRs). The QRDRs investigated consist of the DNA gyrase (*gyrA* and *gyrB*) as well as the DNA topoisomerase IV (*parC and parE*) genes. In our study, only *gyrA* (S83L, D87N, D678E*, A828S*, P872S*) and *parC* genes (S80I, D475E*, T718A* K665R*) were identified. A total of eight isolates harboured both mutations in the QRDR, while one isolate harboured the *gyrA* mutation gene only. Among the identified QRDR mutations, there were known mutations with phenotypic effects known to cause resistance to nalidixic acid and ciprofloxacin (S83L, D87N). Those marked with an asterisk are novel mutations (Table 2).

### 2.3. Sequence Types and Plasmid Replicons

MLST analysis (Table 2) revealed a variety of sequence types (STs), with nine hits from litter and 12 hits from wastewater. These included ST155, 24% (*n* = 6), ST48, 16% (*n* = 4), ST1286, 12% (*n* = 3), and ST602, 8% (*n* = 2), as well as the following singletons: ST3346, 4% (*n* = 1), ST6050, 4% (*n* = 1), ST359, 4% (*n* = 1), ST1771, 4% (*n* = 1), ST6706, 4% (*n* = 1), and ST21, 4% (*n* = 1). The remaining 16% (*n* = 4) of the isolates had unknown STs. All isolates carried plasmid replicons that included IncF, IncFIB, IncFIC, IncFII, IncI2, p0111, IncI1-I(Alpha), IncX1, IncY, Col(pHAD28), IncFIA(HI1), IncFIB(AP001918), IncFIC(FII), IncHI1A, IncHI1B(R27), IncFIA, IncX4, IncQ1, IncB/O/K/Z, IncI(Gamma), Col440I, (pLF82-PhagePlasmid), and ColpVC. The distribution of plasmid replicons among chicken litter and wastewater isolates was even, with Inc being the most common plasmid replicon identified.

### 2.4. Mobile Genetic Elements Associated with ARGs in the Analysed E. coli Genomes

The isolates were further analysed for ARGs and MGEs on NCBI, and two isolates (EC03 and EC11) fell off due to large genomic size, resulting in a total of 23 isolates (Table 3). The main findings from the synteny analysis (Table 3) illustrated the distribution of MGEs across genomes. Almost all ARGs were associated with insertion sequences (*ISs*), except for EC10; others were associated with transposons, except for EC04, EC05, EC07, EC10, EC19, EC25, and EC26, and almost half of the ARGs were carried on integron class 1. A diversity of *ISs* was evident within isolates; two types of transposons, *Tn3* and *TnAs1*, were observed, whereas integrons were represented by intl class 1 only.

### 2.5. Phylogenetic Tree Analysis

**Figure 1:**
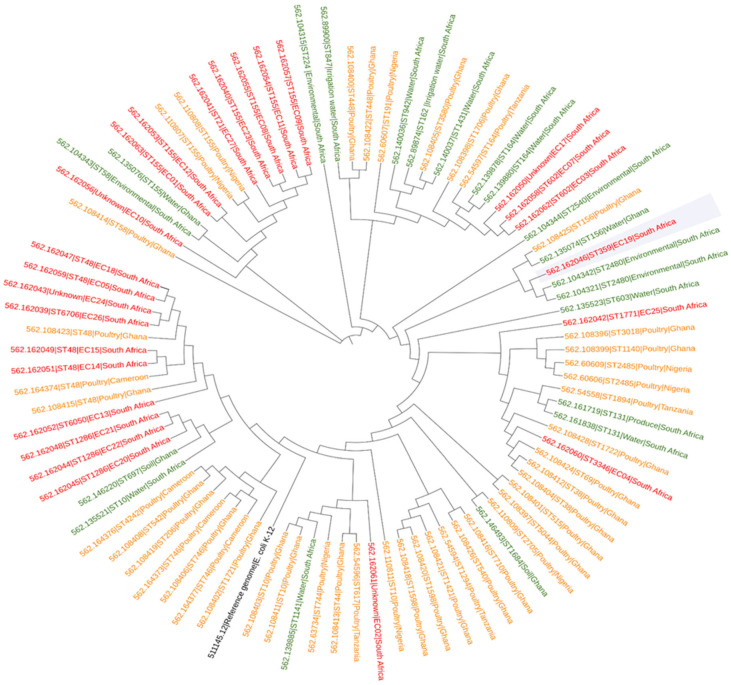
Phylogenetic tree showing *E. coli* isolates from this study (red) alongside poultry (orange) and environmental (green) isolates from various African countries. The phylogenetic tree shows the evolutionary relationships among *E. coli* isolates from different African countries. The construction method can be found on https://www.bv-brc.org/ (accessed on 5 March 2025).

The phylogenetic tree provides insight into the genetic relatedness of *E. coli* isolates from South Africa and various African countries (Figure 1). The *E. coli* genomes from our study showed close genetic relation with poultry-derived *E. coli* from Cameroon, Ghana, Nigeria, and Tanzania; *E. coli* was isolated from the environmental samples, which included unspecified environment isolates, isolates in South Africa, unspecified water from Ghana and South Africa, irrigation water from South Africa, soil from Ghana, and produce from South Africa. These comparisons are based on genomes reported between 2015 and 2024. No data was found for 2025. The isolates appear to have originated from a common ancestor shared with the comparative strains but somehow branched out of the family tree to form new nodes. Multilocus sequence typing (MLST) analysis discovered that a lot of the isolates from our study clustered towards poultry isolates; only a few clustered towards the environmental isolates. Most of the *E. coli* isolates clustered into several sequence types, with ST67, ST48, ST155, and ST164 emerging as the dominant lineages within the intensive poultry production system. A small group of isolates (EC19, EC25, EC04, and EC02) clustered independently from the key poultry-associated *E. coli* cluster in the phylogenetic analysis, highlighting a greater genetic divergence.

### 2.6. Discussion

The study investigated the phenotypic and genomic characteristics of antibiotic-resistant *E. coli* from poultry litter and wastewater from an intensive poultry production system. No presumptive *E. coli* was isolated on all floor swabs sampled after disinfection, indicating an effective sanitation method and less risk of transfer of antibiotic-resistant *E*. *coli* strains from one poultry flock to the next production cycle.

#### 2.6.1. Antibiotic Susceptibility Profile

To better understand resistance trends in poultry-associated *E. coli*, we examined the patterns of antibiotic resistance observed across multiple sampling cycles. The resistance patterns remained largely consistent across the cycles, with all *E. coli* isolates exhibiting relatively high levels of resistance to three antibiotics, i.e., tetracycline (highest prevalence), followed by ampicillin and trimethoprim–sulfamethoxazole. Among the December samples, two isolates (EC02 and EC03) exhibited resistance to ciprofloxacin (Table 1). These findings are consistent with a study by Phiri et al. (10) conducted in Zambia. In that study, broiler litter, cloacal swabs, and carcass swabs were collected from poultry farms, abattoirs, and open markets across seven districts to determine the prevalence and antimicrobial resistance profiles of *Salmonella* and *E. coli*. The *E. coli* isolates demonstrated high resistance to ampicillin (68%), tetracycline (8%), and trimethoprim–sulfamethoxazole (65%), while resistance to ciprofloxacin was comparatively lower (21%). These results are in contrast to the results found by McIver et al. [17] in a study conducted in a similar region to our study, on a commercial poultry farm in South Africa. The study assessed the antibiotic-resistant *E. coli* obtained from litter, faeces, wastewater from the chicken house, truck, crates used in transportation, and final meat products for consumption. The AST results demonstrated a low level of antibiotic resistance to AMP, TET, and STX. In comparison, in this study, the low level of fluoroquinolone resistance observed on ASTs was only evident in two isolates (EC02 and EC03) that were phenotypically resistant to CIP, even though ciprofloxacin ASTs did not correlate with the ARGs identified from the WGS results, where 68% of isolates carried fluoroquinolone-resistant genes. This could have resulted from the fact that under laboratory conditions, resistance genes may be present but not expressed; the presence of a resistant gene in a genome does not always indicate its expression [18].

#### 2.6.2. Antibiotic Resistance and Mobile Genetic Elements

Table 3 reveals that above 98% of isolates were positive for the *qnrS1* gene, which is a plasmid-mediated quinolone resistance (PMQR) gene. Almost all *qnrS1* genes were carried on plasmid, except for EC12 (contig 41), which was carried on chromosome strain 67 (accession number CP128443.1). Antibiotic-resistant genes and MGEs from the *E. coli* were closely related to the target sequence found in a Genbank database (National Library of Medicine, National Center for Biotechnology Information, USA), with the most hits being plasmids (Table 3). Overall, 36% of the isolates carrying the *qnrS1* gene also possessed mutations within the quinolone resistance-determining regions (QRDRs) of the DNA gyrase (*gyrA*) or topoisomerase IV (*parC*) genes. These mutations are known to confer resistance to fluoroquinolones, thereby reducing the effectiveness of this class of antibiotics. Isolates EC03 and EC07 were odd as they exhibited phenotypic resistance that did not correspond with genomic resistance. Isolate EC03 had an antibiogram of CIP-TET-SXT-AMP, and EC07 had TET-SXT-AMP, but both harboured the *fosA3* resistance gene and had a fluoroquinolone point mutation *gyrA* S83L. Isolate EC25 exhibited TET resistance phenotypically, and *aph(6)-Id, aph(3″)-Ib, tet(B)* genomically, but it had a fluoroquinolone point mutation *gyrA* (S83L) and a novel *parC* (K665R).

The isolates’ resistant profile to fluoroquinolones had no correlation between the phenotypic and genomic results, which might have a novel mechanism or carry resistance genes that have not yet been annotated in the database [18]. A large number (68%) of isolates conferring resistance to fluoroquinolones was alarming, as it might render ciprofloxacin ineffective in the future. A review study conducted by [19] revealed that antibiotic-resistant *E. coli* within the poultry industry has increased above 60% globally since 2011 to 2024, with a multidrug resistance surpassing 50%, which is concerning.

Table 3 also demonstrated a wide range of ARGs associated with MGEs, indicating active and varied horizontal gene transfer among *E. coli* strains within the poultry farm. The MGEs identified included class 1 integrons (*intI1*), transposons (*Tn3*, *TnAs1*), and *ISs* (*IS*, *ISKpn19*). Most ARGs were carried; some were associated with one or more MGE. Five ARGs, *dfrA*, *CmlA1*, *ANT(3″)-Ia*, *AadA5*, and *sul3*, were carried on *Intl1*. Most *dfrA-* and *AadA*-resistant genes carried on *intl1* were also associated with *TnAs1*, whereas *ANT(3″)-Ia* was either carried on *intl1* alone or in association with *TnAs1*. This was observed on EC01, EC08, EC10, EC12, EC13, EC24, and EC27. The *DfrA* gene from EC21 and EC22 was carried on *intl1* only. *CmlA1* and *sul3* were carried on *intl1* and associated with *IS256*; refer to EC05, EC18, and EC20. Among the resistant genes harboured by EC02, EC05, and EC20, *QacL* was also identified. This gene encodes proteins found in the Qac (quaternary ammonium compound) efflux pump family, which contributes to antibiotic resistance. EC05 and EC18 also harboured the *esX* (ESAT-6 secretion system)-resistant gene, which confers resistance to macrolides and is known as a virulence factor in *Mycobacterium tuberculosis* [20]. The *esX-resistant* gene is also associated with the opportunistic *M. avium* complex (MAC), which causes a chronic infection of avian tuberculosis in birds. Even though modern husbandry has decreased the incidence of avian tuberculosis within the commercial poultry industry, there have been sporadic outbreaks reported in commercial poultry [21]. Another study conducted by Ogundare et al. [22] in South Africa, Gauteng and Limpopo provinces, aimed to determine the virulence profiles and AMR genes of zoonotic APEC, focusing on foodborne EHEC isolated from close human contacts, poultry, swine, and environmental water samples collected from abattoirs and poultry farms, and it also identified the *esX* gene on their isolates. This might indicate horizontal gene transfer.

#### 2.6.3. Genetic Relatedness

The phylogenomic analysis from our study revealed that the majority of isolates in this study showed closer genetic similarity to poultry-derived *E. coli* than to environmental isolates. The isolates were closely related to poultry *E. coli* isolates found in Cameroon, Ghana, Nigeria, and Tanzania and environmental *E. coli* strains previously identified in South Africa and Ghana (Figure 1). A high number of isolates were closely related to poultry isolates from Ghana and Nigeria, whereas a few exhibited genetic relatedness to isolates circulating in Tanzania. Isolates from our study, such as EC15 (562.162049), EC18 (562.162047), had MLST (ST48), were similar to poultry isolates found in Ghana (562.108415) (562.108423) and Cameroon (562.164374), whereas ST155 was shared by isolates EC08 (562.162055) and EC23 (562.162040), as well as isolates from Nigeria poultry (562.110807 and 562.110808). ST48 is commonly associated with foodborne diseases, mostly prevalent in poultry meat [23]. ST155 is also prevalent in poultry; strains carrying ST155 often harbour different antibiotic resistance, like tetracycline, sulfonamides, and β-lactams [24]. ST155 (24%) was the most common sequence type in our study, followed by ST1286 (12%) and ST602 (8%). All ST155 isolates carried the *tet(A)*-resistant gene, which aligns with findings by Davies et al. [25], who similarly reported a high prevalence of ST155 and the associated presence of the *tet(A)*-resistant gene in *E. coli* from poultry in Bangladesh. The genetic relatedness observed among poultry *E. coli* suggests possible transmission.

## 3. Materials and Methods

### 3.1. Ethical Clearance

Ethical approval was obtained from the University of KwaZulu-Natal Animal Research Ethical Committee (AREC) (reference number AREC00002891/2021). Additionally, a Section 20A permit for researching animals was secured from the Department of Agriculture, Land Reform and Rural Development (DALRRD) (reference number 12/11/1/5 (2283AC)).

### 3.2. Study Site and Population

The study was conducted at an intensive poultry production system located in the uMgungundlovu District of KwaZulu-Natal, South Africa. The farm comprised 12 chicken houses, each housing over 25,000 broiler chickens. One chicken house was randomly chosen as the study site. Samples were collected at the end of each production cycle over three consecutive months: November 2023 for cycle one, December 2023 for cycle two, and January 2024 for cycle three.

Each cycle consisted of 35 days of chicken growth, followed by two days of chicken litter removal (samples of the chicken litter were collected before its removal), one day of chicken house washing (wastewater samples collected on the day of the wash), and one day for disinfecting (floor swab samples were collected 24 h after disinfection).

### 3.3. Sample Collection

A composite sampling strategy was carried out at the end of five weeks, after reaching a full growth production cycle. The chicken house was divided into three rows, and five chicken litter samples were collected from each row at approximately 5 m intervals, starting from the front and moving to the back of the house. This resulted in 15 chicken litter samples. To collect the faecal samples, a sterile disposable spatula and sterile zip-lock bags were used. The samples were stored on ice and transported to the Antimicrobial Resistance Unit (ARU), University of KwaZulu-Natal, Westville campus, for processing.

After two days, based on the hygiene maintenance program of the production, wastewater from the first wash was collected from the drain that allows water flow from inside the chicken house, before mixing with wastewater from the rest of the farm. Two wastewater samples were collected in a 2 L sterile bottle at 30 min time intervals each. Following 24 h of disinfecting the chicken house, the floor was swabbed using sterile swabs, which included swabbing the corners and the drains inside the house. Each sampling point was separated by five steps; the floor swab samples resulted in 75 swabs, which were then placed in one sterile bottle containing sterile distilled water (dH_2_O), making one sample per sampling cycle. Both wastewater and floor swabs followed the same storage and transportation procedure as previously described.

### 3.4. Isolation and Identification of E. coli

*Escherichia coli* was isolated and identified using a method previously described by Mbang et al. [26] with modifications [27].

For chicken litter, 1 g of solid litter was added to 9 mL sterile dH_2_O, briefly vortexed, and filtered. Wastewater was thoroughly mixed and filtered prior to use, and floor swab samples were placed on a shaker at 125 rpm for 2 h before further processing. A one in 100-fold dilution was conducted, where 1 mL of each sample was diluted into 100 mL of sterile distilled water. Subsequently, the putative presence of *E. coli* was determined using the Colilert system (IDEXX Ltd., Westbrook, ME, USA) according to the manufacturer’s instructions, with additional modification (1 g of chicken litter was mixed with 9 mL dH_2_O, filtered before using it in the Colilert system). A single Colilert reagent was added to each sample and mixed well before pouring the mixture onto a Colilert tray that was sealed and incubated at 37 °C for 18–24 h. After incubation, the trays were viewed under a UV light visualiser (Sigma-Aldrich, Steinheim, Germany) at 260 nm. The wells with blue fluorescence indicated putative *E*. *coli* presence. A random selection of wells that fluoresced from each tray was streaked onto a selective medium, Eosin Methylene Blue agar (EMB) (Merck, Darmstadt, Germany), for putative phenotypic identification and incubated at 37 °C for 18–24 h for each sample type. After incubation, two isolates were sub-cultured from EMB onto Nutrient agar (Oxoid, Hampshire, UK) to confirm culture purity and stored in Tryptic Soy Broth (Oxoid, Hampshire, UK) with 20% glycerol at −80 °C for further analysis. Each cycle provided 30 isolates from chicken litter, resulting in a total of 90 isolates for three sampling cycles. For wastewater, each sampling cycle resulted in 2 × 2 L samples that provided 20 isolates, resulting in 60 isolates for three consecutive sampling cycles. A total of 150 presumptive *E. coli* from chicken litter and wastewater were subjected to q-PCR for confirmation. No *E. coli* growth was isolated from floor swab samples. *E. coli* ATCC 25922 (Oxoid, Hampshire, UK) was used as a positive control.

### 3.5. Molecular Confirmation of Isolates

DNA was extracted using the heat lysis method as previously described by Abrar et al., [28]. The extracted DNA was utilised to confirm *E. coli* using the *uidA* gene on a QuantiStudio 5 RealTime PCR System (ThermoFisher Scientific, Waltham, MA, USA) [29] using the forward primer 5′-AAAACGGCAAGAAAAAGCAG-3′ and the reverse primer 5′-ACGCGTGGTTAACAGTCTTGC-3′, which targeted the *uidA* gene. All primers were acquired from Inqaba Biotechnical Industries (Pty) Ltd., Pretoria, South Africa. Optimised thermal cycling conditions for *uidA* (β-D-glucuronidase) included initial uracil–DNA glycosylase (UDG) activation at 50 °C for 2 min, activation of Dual-LockTM polymerase at 95 °C for 2 min, denaturation set at 95 °C for 15 s, annealing set at 60 °C for 15 s, and extension at 72 °C for 10 s for 35 cycles, followed by a final extension at 72 °C for 5 min. A high-resolution melting curve was generated by ramping up the temperature from 65 °C to 95 °C at a continuous rate of 0.15 °C/s. Each reaction included a positive control, *E. coli* ATCC 25922, as well as a negative template control, which consisted of nuclease-free water replacing the DNA template.

### 3.6. Antibiotic Susceptibility Testing (AST)

Confirmed *E. coli* isolates (*n* = 70), 47% were tested against a panel of ten antibiotics using the Kirby–Bauer disc diffusion method on Mueller–Hinton Agar (Oxoid, Hampshire, UK), following the Clinical and Laboratory Standards Institute (CLSI) guidelines [30]. The selection of ten antibiotics aimed to determine whether *E. coli* from the sampling site showed resistance to 3rd and 4th generation cephalosporins, carbapenems, and quinolones, classified as “shared class of antibiotics by animals and humans and also assess the state of antibiotics commonly used by the farm, e.g., ampicillin, trimethoprim-sulfamethoxazole, tetracycline [31]. The antibiotics tested were ampicillin (AMP, 10 μg), ceftriaxone (CRO, 30 µg), cefotaxime (CTX, 30 µg), cefepime (FEP, 30 µg), ceftazidime (CAZ, 30 µg) (CIA), ciprofloxacin (CIP, 5 µg), gentamicin (GEN, 10 µg), meropenem (MEM, 10 µg), tetracycline (TET, 30 µg), and trimethoprim–sulfamethoxazole (SXT, 25 µg). The diameters of the zones of inhibition were measured and interpreted using the breakpoint criteria provided by the CLSI (2020) [30]. *E. coli* ATCC 25922 was used as a positive control.

Out of the 70 confirmed *E. coli* isolates, 25 were selected for whole genome sequencing based on unique antibiograms and MDR profiles, along with two additional isolates that exhibited resistance to tetracycline only. The selection process accounted for all sampling site representation and the three sampling cycles.

### 3.7. Whole Genome Sequencing Analysis and Bioinformatic Analysis

Whole genome sequencing was conducted at the National Institute for Communicable Diseases (NICD), Johannesburg, South Africa. Genomic DNA from bacterial samples was extracted using the GenElute Bacterial Genomic DNA Kit (Sigma Aldrich, St. Louis, MO, USA) according to the manufacturer’s instructions. DNA concentration and purity were assessed at a 260/280 nm absorbance ratio using a Nanodrop 8000 (Thermo Scientific, Waltham, MA, USA). Subsequently, libraries were prepared using the Nextera XT DNA Library Preparation Kit (Illumina, San Diego, CA, USA) and subjected to whole genome sequencing on an Illumina MiSeq platform (Illumina, San Diego, CA, USA). Raw sequence reads were quality-trimmed using Sickle v1.33 (https://github.com/najoshi/sickle, accessed on 15 November 2024) and assembled using the SPAdes v3.6.2 genome assembler. The resulting genomes were submitted to GenBank and assigned accession numbers under BioProject PRJNA1183844. Genome annotation and analysis included identification of antibiotic-resistant genes (ARGs) and disinfectant genes using ResFinder v4.6.0 (https://cge.food.dtu.dk/services/ResFinder, accessed on 16 November 2024). Plasmid replicon typing was obtained via PlasmidFinder v2.1 (https://cge.food.dtu.dk/services/PlasmidFinder/ accessed on 16 November 2024). Multilocus sequence typing (MLST) was performed using the MLST 2.0 database (https://cge.cbs.dtu.dk/services/MLST/; accessed on 17 November 2024.

Mutations conferring resistance to fluoroquinolones and plasmid/chromosomal sequences with the closest nucleotide homology were analysed using BLAST (https://blast.ncbi.nlm.nih.gov/Blast.cgi?PAGE_TYPE=BlastSearch; accessed on 29 July 2025). The DNA gyrase (*GyrA* and *GyrB*) as well as the DNA topoisomerase IV (*parC* and *parE*) genes were analysed on BLAST using the *E. coli* ATCC 25922 strain as a reference. Finally, genome synteny and mobile genetic elements (MGEs) were examined on the National Center for Biotechnology Information (NCBI) platform (https://www.ncbi.nlm.nih.gov/, accessed on 6 January 2025).

### 3.8. Phylogenomic Analyses

A phylogenetic analysis was performed to investigate the relationships among *E. coli* genomes from this study and those reported in South Africa and other African countries from 2015 to 2024. The genomes were downloaded, annotated, and analysed from the Bacterial and Viral Bioinformatics Resource Centre (BV-BRC) (https://www.bv-brc.org/, accessed on 21 September 2025). The comparison included genomes derived from both poultry litter (*n* = 11) and wastewater (*n* = 12). The phylogenetic tree was constructed using the maximum likelihood method on BV-BRC. *E. coli* K-12 (accession: 511145.12) served as a genome reference. Tree annotation, editing, and visualisation were carried out using the Interactive Tree of Life (iTOL) platform (https://itol.embl.de/; accessed on 5 March 2025).

## 4. Conclusions

This study provides genomic insight into antibiotic-resistant *Escherichia coli* circulating within an intensive poultry production system in the uMgungundlovu District, KwaZulu-Natal, South Africa. Antibiotic-resistant *E. coli* were detected in samples from different sources, including faecal matter and wastewater, but they were not recovered from floor surfaces following disinfection. This suggests that the sanitation practices used were effective in reducing *E. coli* contamination on floors.

Findings from faecal matter and wastewater samples revealed a high prevalence of antibiotic-resistant poultry-associated *E. coli*. The isolates harboured multiple antibiotic resistance genes (ARGs) conferring resistance to commonly used classes of antibiotics. The detection of plasmid-mediated resistance genes, together with mutations in quinolone resistance-determining regions, indicates that both acquired and chromosomal mechanisms contribute to the observed resistance profiles.

Furthermore, the presence of diverse mobile genetic elements in poultry-derived *E. coli* highlights the potential for horizontal gene transfer within the poultry production environment. Overall, the results demonstrate that *E. coli* from intensive poultry systems are multidrug-resistant and carry numerous transmissible ARGs. The release of these resistant bacteria into the environment through faecal waste and wastewater following production cycles represents a significant public health concern.

## 5. Study Limitations

This study provides valuable genomic insight into antibiotic-resistant *Escherichia coli* within an intensive poultry production system; however, several limitations should be acknowledged. First, sampling was restricted to a single chicken house within one production system, which may limit the generalisability of the findings to other poultry farms, production systems, or geographic regions. Second, the study represents a snapshot in time, and the absence of time-resolved sampling limits the ability to assess temporal dynamics of antimicrobial resistance emergence, persistence, and dissemination across production cycles.

In addition, while isolates were collected from multiple sample types, detailed spatial metadata within the production environment was limited. This constrains robust inference regarding transmission pathways and the directionality of bacterial spread between poultry, waste streams, and the surrounding environment. Finally, although genomic analyses identified resistance-associated mutations, including a potentially novel mutation, functional validation was not performed, and, therefore, the phenotypic contribution of these mutations to antimicrobial resistance remains to be confirmed.

## Figures and Tables

**Table 1:** Antibiotic susceptibility profiles of *E. coli* from a commercial poultry farm.

	November Isolates (*n* = 27)			December Isolates (*n* = 26)			January Isolates (*n* = 17)			Total Susceptibility (*n* = 70)		
Antibiotic Agent	S	I	R	S	I	R	S	I	R	S	I	R
GEN	24	3	0	24	2	0	16	1	0	64	6	0
AMP	1	1	25	1	0	25	1	0	16	3	1	66
MEM	27	0	0	26	0	0	17	0	0	70	0	0
CTX	27	0	0	26	0	0	17	0	0	70	0	0
CAZ	27	0	0	26	0	0	17	0	0	70	0	0
CRO	27	0	0	26	0	0	17	0	0	70	0	0
FEP	27	0	0	26	0	0	17	0	0	70	0	0
CIP	21	6	0	18	6	2	13	4	0	52	16	2
SXT	7	1	19	6	0	20	2	1	14	15	2	53
TET	0	0	27	0	0	26	0	0	17	0	0	70

Abbreviations: GEN, gentamicin; AMP, ampicillin; MEM, meropenem; CTX, cefotaxime; CAZ, ceftazidime; CRO, ceftriaxone; FEP, cefepime; CIP, ciprofloxacin; SXT, trimethoprim–sulfamethoxazole; TET, tetracycline; S, susceptible; I, intermediate; R, resistant.

**Table 2:** Phenotypic and genomic analysis of *E. coli* isolates collected from chicken litter and wastewater from an intensive poultry production system in uMgungundlovu district, South Africa.

Isolate ID	Source	Sequence Type	Resistance Pattern	ARGs	Disinfect. Gene	Plasmid Replicon	Fluoroquinolone Point Mutations			
							*gyrA*	*gyrB*	*parC*	*parE*
EC01	Litter	ST155	TET-SXT-AMP	*aadA5*, *qnrS1*, *sul2*, *ul3*, *tet(A)*, *dfrA14*, *dfrA17*, *bleO*, *bla_TEM-1B_*	No hit	*IncFIB*, *IncFII*, *IncI2*, *p0111*	-	-	-	-
EC02	Litter	Unknown	CIP-TET-AMP	*aph(3′)-Ia*, *ant(3″)-Ia*, *aadA2*, *cmlA1*, *sul2*, *sul3*, *tet(A)*, *dfrA12*, *bleO*, *bla_TEM-1B_*	*sitABCD*	*IncFIB*, *IncFIC*, *IncI1-I(Alpha)*, *IncX1*	S83L, D87N	-	S80I	-
EC03	Litter	ST602	CIP-TET-SXT-AMP	*fosA3*	*sitABCD*		S83L	-	-	-
EC04	Litter	ST3346	TET-SXT-AMP	*aph(6)-Id*, *aph(3″)-lB*, *qnrB19*, *tet(B)*	*sitABCD*	*IncFIB*, *IncY*, *Col(pHAD28)*, *ColpVC*	D678E* A828S*	-	D475E* T718A*	-
EC05	Wastewater	ST48	TET-SXT-AMP	*ant(3″)-Ia*, *aadA2b*, *bla_LAP-1_*, *fosA4*, *cmlA1*, *qnrS1*, *sul3*, *tet(B)*	*sitABCD*	*IncFIA(HI1)*, *IncFIB(AP001918)*, *IncFIC(FII)*, *IncFII*, *IncHI1A*, *IncHI1B(R27)*, *IncI2*, *p0111*	-	-	-	-
EC07	Litter	ST602	TET-SXT-AMP	*fosA3*	*sitABCD*	*IncFIA*, *IncFIB(AP001918)*, *IncFII*	S83L	-	-	-
EC08	Litter	ST155	TET-SXT-AMP	*aadA5*, *qnrS1*, *tet(A)*, *sul2*, *dfrA17*, *bleo*	*sitABCD*	*ColpVC*, *IncFIB(AP001918)*, *IncFIC(FII)*, *IncX4*	-	-	-	-
EC09	Wastewater	ST155	TET-SXT-AMP	*aadA5*, *qnrS1*, *tet(A)*, *dfrA17*, *bleO*	*sitABCD*	*ColpVC*, *IncFIB(AP001918)*, *IncFIC(FII)*, *IncX4*	-	-	-	-
EC10	Litter	Unknown	TET-SXT-AMP	*aph(6)-Id*, *aph(3”)-lb*, *sul2*, *tet(A)*, *dfrA5*, *bla_TEM-1B_*	*sitABCD*	*IncFIB(AP001918)*, *IncFII*, *IncI2*, *IncQ1*, *p0111*	-	-	-	-
EC11	Wastewater	ST155	TET-SXT-AMP	*aadA5*, *qnrS1*, *sul2*, *tet (A)*, *dfrA17*, *bleO*	*sitABCD*	*ColpVC*, *IncFIB(AP001918)*, *IncFIC(FII)*, *IncX4*	-	-	-	-
EC12	Wastewater	ST155	TET-SXT-AMP	*aadA5*, *qnrS1*, *sul2*, *sul3*, *tet (A)*, *dfrA14*, *dfrA17*, *bleO*, *bla_TEM-1B_*	No hit	*IncFIB(AP001918)*, *IncFII*, *IncX1*	-	-	-	-
EC13	Litter	ST6050	TET-AMP	*aadA1*, *aadA2*, *aph(6)-Id*, *aph(3”)-la*, *cml1*, *qnrS1*, *sul3*, *tet (A)*, *dfrA12*, *dfrA14*, *bleO*	No hit	*IncFIB(AP001918)*, *IncI1-I(Alpha)*, *IncX1*, *p0111*	S83L P872S*	-	S80I	-
EC14	Litter	ST48	TET-AMP	*aph(3′)-Ia*, *OqxA*, *OqxB*, *tet (A)*, *bleO*, *bla_TEM-135_*,	*sitABCD*	*ColpVC*, *IncB/O/K/Z*, *IncX1*, *p0111*	S83L	-	S80I	-
EC15	Wastewater	ST48	TET-SXT-AMP	*aph(3′)-Ia*, *OqxA*, *OqxB*, *tet(A)*, *bleO*, *bla_TEM-135_*	*sitABCD*	*ColpVC*, *IncB/O/K/Z*, *IncX1*, *p0111*	S83L	-	S80I	-
EC17	Wastewater	Unknown	TET-SXT	*ant(3″)-Ia*, *fosA3*, *cmlA1*, *qnrS1*, *sul3*, *tet(A)*, *dfrA15*, *bla_TEM-135_*	*sitABCD*	*IncFIA*, *IncFIB(AP001918)*, *IncFIC(FII)*, *IncFII*	-	-	-	-
EC18	Wastewater	ST48	TET-AMP	*tet (A)*, *tet (M)*, *bla_TEM-1B_*	No hit	*IncFIA*, *IncFIB(AP001918)*, *IncFII*, *IncI(Gamma)*, *IncX1*	-	-	-	-
EC19	Wastewater	ST359	TET	*aadA1*, *ant(3″)-Ia*, *aph(3′)-Ia*, *cmlA1*, *sul3*, *tet(A*, *sul3*, *tet(A)*, *dfrA12*, *bleO*	*sitABCD*	*IncFIB(AP001918)*, *IncFIC(FII)*, *IncI1-I(Alpha)*, *IncI2*	S83L, D87N	-	S80I	-
EC20	Wastewater	ST1286	TET-SXT-AMP	*aph(3′)-Ia*, *qnrS1*, *OqxA*, *OqxB*, *sul2*, *dfrA14*, *bleO*	No hit	*IncFIB(AP001918)*, *IncFII*, *IncI1-I(Alpha)*, *IncX1*	-	-	-	-
EC21	Wastewater	ST1286	TET-SXT-AMP	*aph(3″)-la*, *qnrS1*, *OqxA*, *OqxB*, *sul2*, *dfrA14*, *bleO*	No hit	*IncFIB(AP001918)*, *IncFII*, *IncI1-I(Alpha)*, *IncX1*	-	-	-	-
EC22	Litter	ST1286	TET-AMP	*aph(3′)-la*, *qnrS1*, *OqxA*, *OqxB*, *sul2*, *dfrA14*, *bleO*	No hit	*ColpVC*, *IncFIB(AP001918)*, *IncFII*, *IncI1-I(Alpha)*, *IncX1*	-	-	-	-
EC23	Litter	ST155	TET-SXT	*aadA5*, *qnrS1*, *sul2*, *tet (A)*, *dfrA17*, *bleO*	*sitABCD*	*ColpVC*, *IncFIB(AP001918)*, *IncFIC(FII)*, *IncX4*	-	-	-	-
EC24	Litter	Unknown	TET-AMP	*aph(6)-Id*, *fosA3*, *qnrS1*, *tet(A)*, *bleO*, *bla_TEM-1B_*	*sitABCD*	*Col440I*, *IncFIB(AP001918)*, *IncFIC(FII)*, *IncFII(29)*, *IncI1-I(Alpha)*, *IncN*, *IncX1*, *p0111*	S83L	-	K665R*	-
EC25	Wastewater	ST1771	TET	*aph(6)-Id*, *aph(3″)-Ib*, *tet(B)*	*sitABCD*	*IncFIB(AP001918)*, *IncFIB(pLF82-PhagePlasmid)*, *IncFII*	S83L	-	K665R*	-
EC26	Wastewater	ST6706	TET-AMP	*aph(6)-Id*, *qnrS1*, *tet(A)*, *bleO*	*sitABCD*	*Col440I*, *IncFIB(AP001918)*, *IncFIC(FII)*, *IncX1*, *p0111*	-	-	-	-
EC27	Wastewater	ST21	TET-AMP	*aadA5*, *qnrS1*, *tet(B)*, *tet(A)*, *dfrA17*, *bleO*	*sitABCD*	*Col440I*, *IncFIB(AP001918)*, *IncFIC(FII)*, *IncX1*, *p0111*	-	-	-	-

*: Novel fluoroquinolones point mutations.

**Table 3:** Genetic environment of antibiotic resistance genes and mobile genetic elements in *E. coli* from poultry in uMgungundlovu.

Isolate ID	Source	Contig	Synteny of Antibiotic Resistance Genes and MGEs	Plasmid/Chromosomal Sequence with the Closest Nucleotide Homology (Accession Number)
EC01	Litter	87	*ANT(3″)-Ia*:*ANT(3″)-Ia*:*DfrA17*	*Escherichia coli* isolate A21005 plasmid pA21005_D, complete sequence (CP185942.1)
		53	*tet(A)*:*tetR(A)*:transposase::recombinase: ISKpn19: recombinase:*QnrS1*:*IS3*:*Tn3*	*Escherichia coli* strain LA058 plasmid pLAO31, complete sequence (OP242261.1)
		79	*sul2*::*IS5075*	*Escherichia coli* strain CFS3313 plasmid pCFS3313-2, complete sequence (CP026941.2)
		86	*Bla_TEM-1_*:recombinase	*Escherichia coli strain* ExPEC_A376 plasmid pA376_p0, complete sequence (CP142553.1)
		69	*dfrA14*:*intI1*::recombinase family protein: *TnAs1*	*Escherichia coli* strain 2021CK-01361 plasmid unnamed1 (CP107721.1)
EC02	Litter	20	*aph(3′)-Ia*::*Tn3*	*Escherichia coli* strain F5 plasmid p2, complete sequence (CP195957.1)
		272	*IS256*:*QacL*:*ANT(3″)-Ia*:*CmlA1*:*ANT(3″)-Ia*:*dfrA12*:*intI1*	*Escherichia coli* strain CP61_Sichuan plasmid pCP61-IncFIB, complete sequence (CP053729.1)
		28	*Sul2*::transposase::*IS1*	*Escherichia coli* strain 746 chromosome, complete genome (CP023353.1)
		485	tet(A):TetR(A):	*Escherichia coli* strain PNCE004196 plasmid pH2S-IncY, complete sequence (CP195148.1)
		555	*blaT_EM-1_*:*recombinase*	*Escherichia coli* strain GDSC2BY4G plasmid pHNBY4G-1, complete sequence (CP136026.1)
EC04	Litter	38	*aph(6)-Id”*:*aph(3″)-Ib*:*IS1133*:recombinase::*IS4*	*Escherichia coli* strain L-I1 plasmid pIncFIB, complete sequence (MH422552.1)
		42	Transposase:Tet(C):tet(B):tetR(B)	*Escherichia coli* strain A31232 plasmid p2_A31232, complete sequence (CP181649.1)
EC05	Wastewater	78	sul3::IS256:ANT(3″)-Ia:CmlA1:ANT(3″)-Ia:HAD family hydrolase:estX:intI1	*Escherichia coli* strain RHB02-E1-C06 plasmid unnamed1, complete sequence (CP099300.1)
		57	*QnrS1*:IS3: *bla_LAP-1_*:ISKpn19:recombinase	*Escherichia coli* strain EC6622 plasmid pEC6622-2, complete sequence (CP096589.1)
		51	*Tet(C)*:*tet(B)*:*tetR(B)*	*Escherichia coli* strain ET120 plasmid unnamed1, complete sequence (CP101002.1)
EC07	Litter	67	*IS6*:*FosA3*: *TetR*	*Escherichia coli* strain AH01 plasmid pAH01-3, complete sequence (CP055254.1)
EC08	Litter	67	aadA5:*dfrA17*: *intI1*::recombinase:TnAs1	*Escherichia coli* isolate J31 plasmid pJ31, complete sequence (CP053788.1)
		41	*QnrS1*:Recombinase: ISKpn19:recombinase::transposase:*tetR(A)*:*tet(A)*::*TnAs1*	*Escherichia coli* strain 67 chromosome (CP128443.1)
		46	*ISVsa3*::*Sul2*	*Escherichia coli* strain CDF6 chromosome, complete genome (CP158429.1)
		71	*Tn3*::*bleO*	*Escherichia coli* strain PE15 plasmid pPE15-IncF, complete sequence (CP041629.1)
EC09	Wastewater	65	*tet(A)*:*tetR(A)*:Transposase::Recombinase:ISKpn19:Recombinase:*QnrS1*:	*Escherichia coli* strain GD-33 plasmid pNDM33-1, complete sequence (MN915011.1)
		73	*IS26*::*Tn3*:*bleO*	*Escherichia coli* strain ETEC1722 plasmid unnamed5, complete sequence (CP122849.1)
EC10	Litter	58	*aph(6)-Id*: *aph(3″)-Ib”*:*Sul2*	*Escherichia coli* strain CFS3313 plasmid pCFS3313-1, complete sequence (CP026940.2)
		61	*tet(A)*:*tetR(A)*:relaxase	*Escherichia coli* strain JCKP02 plasmid p-1122, complete sequence (CP195760.1)
		68	Transposase:*bla_TEM-1_*:Recombinase	*Escherichia coli* O168:H8 OkiPb01715 plasmid pOkiPb01715_2 DNA, complete sequence (AP042697.1)
		26	*TnAs1*:Recombinase::*intI1*:*DfrA5*	*Escherichia coli* strain GN02461 plasmid p2461-1 (CP095535.1)
EC12	Wastewater	67	*AadA5*:*dfrA17*:*intI1*::recombinase:*TnAs1*	*Escherichia coli* isolate J31 plasmid pJ31, complete sequence (CP053788.1)
		41	*QnrS1*:recombinase:ISKpn19:recombinase::transposase:*tetR(A)*:*tet(A)*	*Escherichia coli* strain 67 chromosome (CP128443.1)
		73	*sul2*::*IS5075*	*Escherichia coli* strain EC-14-2-9 plasmid pEC-14-2-9-2, complete sequence (CP093284.1)
		71	*Tn3*::*bleO*	*Escherichia coli* strain PE15 plasmid pPE15-IncF, complete sequence (CP041629.1)
EC13	Litter	75	*IS91*:*tet(O)*:*Tn3*:	*Escherichia coli* strain APEC-O117H42 plasmid pAPEC-O117H42-C, complete sequence (CP172334.1)
		83	*bla_TEM_*::recombinase	*Escherichia coli strain* ECE228 plasmid unnamed2, complete sequence *(CP196314.1)*
		31	*AadA5*:*dfrA17*:*intI1*::*recombinase*:*TnAs1*	*Escherichia coli* strain NCTC11129 genome assembly, chromosome: 1 (LR134222.1)
		54	*tet(A)*:*tetR(A)*:transposase::recombinase:ISKpn19:recombinase:*QnrS1*:*IS3*:*Tn3*	*Escherichia coli* strain LA058 plasmid pLAO31, complete sequence (OP242261.1)
		76	*bla_TEM_*:*sul3*:	*Escherichia coli* strain WTP03 plasmid pWTP-03, complete sequence (OR287787.1)
		78	*Sul2*::*IS5075*	*Escherichia coli strain* CFS3313 plasmid pCFS3313-2, complete sequence (CP026941.2)
EC14	Litter	88	*IS5*:*IS903B*:*aph(6)-I*	*Escherichia coli* strain EC24 plasmid unnamed2, complete sequence (CP182193.1)
		109	*bla_TEM1_*:recombinase:*IS256*	*Escherichia coli* strain GDSC2BY4G plasmid pHNBY4G-1, complete sequence (CP136026.1)
		82	ISKpn19:recombinase:QnrS1:IS3:Tn3	*Escherichia coli* strain LD91-1 plasmid pLD91-1, complete sequence (CP192622.1)
		78	*IS256*:*QacL*:*ANT(3″)-Ia*:*CmlA1*:*ANT(3″)-Ia*::*DfrA12*	*Escherichia coli* strain CP61_Sichuan plasmid pCP61-IncFIB, complete sequence (CP053729.1)
		107	*tet(A)*:*tetR(A)*	*Escherichia coli* strain C3728 plasmid p1, complete sequence (CP196348.1)
EC15	Wastewater	51	*aph(3′)-Ia*::Tn3	*Escherichia coli* strain F5 plasmid p2, complete sequence (CP195957.1)
		118	*Tn3*:*bla_TEM135_*:recombinase:	*Escherichia coli* strain elppa2 plasmid unnamed2, complete sequence (CP083532.1)
		100	IS6::oqxB:OqxA	*Escherichia coli* strain FS11Y5C plasmid pFS11Y5CT, complete sequence (MG014721.1)
		101	*TnAs1*:::*tet(A)*:*tetR(A)*:Transposase	*Escherichia coli* strain I3 plasmid p2, complete sequence (CP195971.1)
		125	*IS26*:*bleO*	*Escherichia coli* strain GDSC2BY4G plasmid pHNBY4G-1, complete sequence (CP136026.1)
EC17	Wastewater	45	Recombinase:bla_TEM-135_:Tn3:Transposase:*TetR(A)*:*Tet(A)*:::TnAs1: Recombinase:::*IS903B*::::::::::::::::::::::::::*aph(3′)-Ia*::*Tn3*	*Escherichia coli* strain PEC027 plasmid pEC027-2, complete sequence (CP195927.1)
		95	*OqxB*:*OqxA*	*Escherichia coli* strain FS11Y5C plasmid pFS11Y5CT, complete sequence (MG014721.1)
EC18	Wastewater	60	*intI1*:*estX*:HAD family hydrolase:*CmlA1*:*ANT(3″)-Ia*: *QacL*:*IS256*::*sul3*	*Escherichia coli* strain RH-024-WU chromosome *(CP050201.1)*
		516	Relaxase:*Tn3*:*bla_TEM-1__35_*:*recombinase*:	*Escherichia coli* strain PE143 genome assembly, plasmid: pPE143_2 (OZ249096.1)
		580	IS6:FosA3:	*Escherichia coli strain AH01 plasmid pAH01-3*, *complete sequence (CP055254.1)*
		127	*IS6*:ISKpn19:recombinase:*QnrS1*:*IS3*:*Tn3*:	*Escherichia coli* strain PE143 genome assembly, plasmid: pPE143_2 (OZ249096.1)
		334	*Tet(A)*:*TetR(A)*	*Escherichia coli strain C3728 plasmid p1*, *complete sequence (CP196348.1)*
EC19	Wastewater	67	*Tet(A)*:*TetR(A)*:transposase::recombinase: ISKpn19	*Escherichia coli* strain F5 plasmid p2, complete sequence (CP195957.1)
		80	*Tet(M)*::*IS256*: integron integrase:*IS5*	*Escherichia coli* FUJ80155 plasmid pFUJ80155-1 DNA, complete sequence (AP024695.1)
		92	*Bla_TEM-1_*:recombinase	*Escherichia coli* O111:H8 strain 7-55 62A plasmid p7_55_62A-2, complete sequence (CP077508.1)
EC20	Wastewater	55	*sul3*::*IS256*:*QacL*:ANT(3″)-Ia:CmlA1:ANT(3″)-Ia::*DfrA12*: *intI1*	*Escherichia coli* strain RW8-1 plasmid pRW8-1_122k_tetX, complete sequence (MT219826.1)
		92	*Tet(A)*:*TetR(A)*	*Escherichia coli* strain S3 plasmid pCol156-IncF-rep2131, complete sequence (CP196492.1)
		64	*Tn3*:*bleO*:ISKpn26:	*Escherichia coli* strain SFE8 chromosome, complete genome (CP051219.1)
EC21	Wastewater	47	*aph(3′)-Ia*::*Tn3*:::*IS6*	*EEscherichia coli* strain F5 plasmid p2, complete sequence (CP195957.1)
		85	Relaxase::recombinase:ISKpn19:Recombinase:*QnrS1*:Transposase	*Escherichia coli* strain GD-33 plasmid pNDM33-1, complete sequence (MN915011.1)
		86	*oqxB*:*oqxA*	*Escherichia coli* strain C21 plasmid pC21-2, complete sequence (CP052879.1)
		32	Transposase:*Sul2*::Transposase::*1S1*	*Escherichia coli* strain HNTH2207 chromosome (CP137721.1)
		97	*dfrA14*:*intI1*	*Escherichia coli* strain ECE228 plasmid unnamed2, complete sequence (CP196314.1)
		90	*bleO*::*Tn3*	*Escherichia coli* strain PE15 plasmid pPE15-IncF, complete sequence (CP041629.1)
EC22	Litter	47	aph(3′)-Ia::Tn3	*EEscherichia coli* strain F5 plasmid p2, complete sequence (CP195957.1)
		89	Recombinase:ISKpn19:Recombinase:*QnrS1*:Transposase	*Escherichia coli* strain GD-33 plasmid pNDM33-1, complete sequence (MN915011.1)
		90	*oqxB*:*oqxA*	*Escherichia coli* strain C21 plasmid pC21-2, complete sequence (CP052879.1)
		28	*Sul2*::Transposase::*ISI*	*Escherichia coli* strain HNTH2207 chromosome (CP137721.1)
		103	*dfrA14*:*intI1*	*Escherichia coli* strain EC15 plasmid pEC15-AU-EG3, complete sequence (CP194960.1)
		96	*bleO*::*Tn3*	*Escherichia coli* strain PE15 plasmid pPE15-IncF, complete sequence (CP041629.1)
EC23	Litter	46	*aph(3′)-Ia*::*Tn3*:::*IS6*	*EEscherichia coli* strain F5 plasmid p2, complete sequence (CP195957.1)
		86	recombinase:ISKpn19:Recombinase:*QnrS1*:Transposase	*Escherichia coli* strain GD-33 plasmid pNDM33-1, complete sequence (MN915011.1)
		88	*OqxB*:*OqxA*	*Escherichia coli* strain C21 plasmid pC21-2, complete sequence (CP052879.1)
		92	*bleO*::*Tn3*	*Escherichia coli* strain PE15 plasmid pPE15-IncF, complete sequence (CP041629.1)
		99	*dfrA14*:*intI1*	*Escherichia coli* strain LD22-1 plasmid pLD22-1-135kb, complete sequence (CP047878.1)
		30	transposase:: *sul2*::Transposase: *IS1*	*Escherichia coli* strain HNTH2207 chromosome (CP137721.1)
EC24	Litter	66	*ANT(3″)-Ia*:*DfrA17*:*intI1*::Recombinase:*TnAs1*	*Escherichia coli* isolate J31 plasmid pJ31, complete sequence (CP053788.1)
		61	*Tet(A)*:*TetR(A)*:Transposase::recombinase::ISKpn19:Recombinase:*QnrS1*:Transposase	*Escherichia coli* strain GD-33 plasmid pNDM33-1, complete sequence (MN915011.1)
		70	*Tn3*::*bleO*	*Escherichia coli* strain PE15 plasmid pPE15-IncF, complete sequence (CP041629.1)
EC25	Wastewater	277	*IS903B*:*aph(6)-I*	*Escherichia coli* strain 58-3 plasmid pCD58-3-1, complete sequence (CP050037.1)
		372	*FosA3*:::*tetR*:DDE integrase	*Escherichia coli* strain AH01 plasmid pAH01-3, complete sequence (CP055254.1)
		55	*bla_TEM-1_*:Recombinase	*Escherichia coli* strain CUVET18-789 plasmid pCUVET18-789.3, complete sequence (CP115315.1)
		453	*QnrS1*:transposase	*Escherichia coli* strain ECE228 plasmid unnamed2, complete sequence (CP196314.1)
EC26	Wastewater	28	*aph(6)-Id*:*aph(3″)-Ib*:*IS1133*:Recombinase::*ISVsa5*	*Escherichia coli* strain MS1665 plasmid pMS1665-1, complete sequence (CP097722.1)
		46	*tetC*:*tet(B)*:*tetR(B)*:	*Escherichia coli* strain EC0880B genome assembly, plasmid: 2 (OX460319.1)
EC27	Wastewater	86	*IS5*::*IS903B*:*aph(6)-I*	*Escherichia coli* strain EC24 plasmid unnamed2, complete sequence (CP182193.1)
		83	ISKpn19:Recombinase:*QnrS1*:*IS3*:*Tn3*	*Escherichia coli* strain ECE228 plasmid unnamed2, complete sequence (CP196314.1)
		344	*ANT(3″)-Ia*:*DfrA17*:*IntI1*::Recombinase:*TnAs1*	*Escherichia coli* isolate J31 plasmid pJ31, complete sequence (CP053788.1)
		172	*tet(A)*:*tetR(A)*:Transposase::Recombinanse:ISKpn19:Recombinase:*QnrS1*:Transposase	*EEscherichia coli* strain GD-33 plasmid pNDM33-1, complete sequence (MN915011.1)
		112	*tetR(B)*:*tet(B)*:*tetC*:*ISVsa5*:*IS4*	*Escherichia coli* strain B1172 chromosome, complete genome (CP120549.1)
		515	*Tn3*::*bleO*	*Escherichia coli* strain PE15 plasmid pPE15-IncF, complete sequence (CP041629.1)

## Data Availability

The data presented in this study are openly available in GenBank at https://www.ncbi.nlm.nih.gov/search/all/?term=PRJNA1183844 (accessed on 21 December 2025).
